# Cultural Adaptation of Minimally Guided Interventions for Common Mental Disorders: A Systematic Review and Meta-Analysis

**DOI:** 10.2196/mental.5776

**Published:** 2016-09-26

**Authors:** Melissa Harper Shehadeh, Eva Heim, Neerja Chowdhary, Andreas Maercker, Emiliano Albanese

**Affiliations:** ^1^ Institute of Global Health Faculty of Medicine University of Geneva Geneva Switzerland; ^2^ Division of Psychopathology and Clinical Intervention Department of Psychology University of Zürich Zürich Switzerland; ^3^ London School of Hygiene and Tropical Medicine (prior affiliation) London United Kingdom; ^4^ Sangath (prior affiliation) Sangath India; ^5^ Department of Psychiatry Faculty of Medicine University of Geneva Geneva Switzerland

**Keywords:** cultural adaptation, depression, anxiety, self-help, minimally guided intervention, e-mental health, bibliotherapy

## Abstract

**Background:**

Cultural adaptation of mental health care interventions is key, particularly when there is little or no therapist interaction. There is little published information on the methods of adaptation of bibliotherapy and e-mental health interventions.

**Objective:**

To systematically search for evidence of the effectiveness of minimally guided interventions for the treatment of common mental disorders among culturally diverse people with common mental disorders; to analyze the extent and effects of cultural adaptation of minimally guided interventions for the treatment of common mental disorders.

**Methods:**

We searched Embase, PubMed, the Cochrane Library, and PsycINFO for randomized controlled trials that tested the efficacy of minimally guided or self-help interventions for depression or anxiety among culturally diverse populations. We calculated pooled standardized mean differences using a random-effects model. In addition, we administered a questionnaire to the authors of primary studies to assess the cultural adaptation methods used in the included primary studies. We entered this information into a meta-regression to investigate effects of the extent of adaptation on intervention efficacy.

**Results:**

We included eight randomized controlled trials (RCTs) out of the 4911 potentially eligible records identified by the search: four on e-mental health and four on bibliotherapy. The extent of cultural adaptation varied across the studies, with language translation and use of metaphors being the most frequently applied elements of adaptation. The pooled standardized mean difference for primary outcome measures of depression and anxiety was -0.81 (95% CI -0.10 to -0.62). Higher cultural adaptation scores were significantly associated with greater effect sizes (*P*=.04).

**Conclusions:**

Our results support the results of previous systematic reviews on the cultural adaptation of face-to-face interventions: the extent of cultural adaptation has an effect on intervention efficacy. More research is warranted to explore how cultural adaptation may contribute to improve the acceptability and effectiveness of minimally guided psychological interventions for common mental disorders.

## Introduction

### Globalization of Minimally Guided Interventions

There is an alarming mismatch between the prevalence of mental disorders and the availability of services to meet mental health needs, particularly in low- and middle-income countries (LMICs) [1]. The Movement for Global Mental Health [2] emphasizes increasing the coverage of treatments for mental disorders worldwide, particularly in countries where the *treatment gap* is greatest (ie, in low- and middle-income countries) [3]. There is a growing interest in how to deliver psychological interventions to diverse populations, and various innovative solutions may expand their reach and accessibility in low- and high-income countries alike.

Evidence shows that minimally guided interventions (ie, self-help and guided self-help) may be as efficacious as face-to-face interventions for the treatment of a broad range of common mental disorders [4], including in routine care [5], with guided self-help being slightly superior to complete self-help [6]. Indeed, the World Health Organization (WHO) has updated recommendations on the treatment of depression in low-resource settings to include both face-to-face (eg, high therapist investment) and self-help interventions [7].

Bibliotherapy (ie, therapeutic books) and e-mental health are established means of providing minimally guided psychological interventions requiring one hour or less of face-to-face support time or up to 90 minutes total telephone or email support [8]. They may also appeal to people who are concerned about stigma associated with accessing mental health services. However, evidence on their efficacy is currently limited to high-income countries (HICs) and in culturally homogenous groups [9,10]. A recent systematic review found only three studies on e-mental health interventions in LMICs [11]. Nevertheless, these types of interventions may be viable solutions to narrow the mental health treatment gap in LMICs [12,13], where two-thirds of the 3.2 billion people using the Internet live [14], and where literacy rates are rapidly rising—currently estimated at 85% of the world population [15]. An important consideration is that in areas with restricted resources and as a means of increasing coverage of minimally guided interventions, the guidance may be given by a trained layperson, such as a family carer [16] or a community volunteer.

### Cultural Adaptation

Intervention developers and care providers should ensure that treatments are suited to the culture of the intended users, both for moral reasons and for technical, efficacy-related reasons [16]. Cultural adaptation is defined as “the systematic modification of an evidence-based treatment or intervention protocol to consider language, culture, and context in such a way that it is compatible with the client’s cultural patterns, meanings, and values” [17]. Cultural adaptation to the needs and expectations of intended users is likely very important for minimally guided interventions because there is little or no therapist interaction to carry the dimension of culture into the intervention.

The Bernal and Sáez-Santiago framework was proposed in 1995 as a framework for planning and conducting interventions with culturally and linguistically diverse (CALD) clients [18,19]. It has eight elements of adaptation: (1) language, (2) person (client) attributes, (3) metaphors, (4) content, (5) concepts, (6) goals, (7) methods, and (8) context of the intervention or services [19].

Evidence suggests that culturally adapted interventions may be more efficacious than interventions that have not been adapted [20], and that effectiveness increases with the number of implemented adaptation elements, according to the Bernal and Sáez-Santiago framework [21,22]. Evidence from systematic reviews show that the extent to which face-to-face interventions are culturally adapted varies considerably [23,24]. However, little is known about the methods and potential benefits of cultural adaptation of minimally guided interventions designed to improve the mental health and well-being of CALD populations.

The aim of this paper was to understand the extent and effects of cultural adaptation of minimally guided interventions for the treatment of common mental disorders. We conducted a systematic review and meta-analysis of experimental studies of minimally guided interventions for the treatment of common mental disorders—depression, anxiety, and adjustment disorders—conducted with culturally diverse populations. We also tested whether the characteristics and extent of cultural adaptation of the minimally guided interventions under study were associated with the combined effect estimates.

## Methods

The methods and procedures used to conduct the systematic review and meta-analysis are reported in accordance with the Preferred Reporting Items for Systematic Reviews and Meta-Analyses (PRISMA) statement [25].

### Systematic Search

We designed our search strategy by combining relevant acronyms and synonyms that captured the population, intervention, comparator, and outcomes (PICO) elements, and the study design consistent with our study aim. Professional librarians from the University of Geneva and the University of Zurich assisted in testing and refining the search strategy, which was written for PubMed and adapted to Embase, the Cochrane Library, and PsycINFO.

We conducted a database search of Embase, PubMed, Cochrane Library, and PsycINFO. Four search concepts were combined in order to capture relevant literature: mode of delivery (eg, mobile phone, multimedia, and Web based), intervention program (eg, self-help, minimally guided, and Internet cognitive behavioral therapy [iCBT]), common mental disorders (eg, depression, anxiety, stress, and trauma), and cultural diversity. In order to identify culturally diverse populations, we took a proxy of LMICs classified according to the World Bank [26], using their names and population adjectives with additional high-income country and population names that we considered to be culturally divergent from North America, Europe, and Australia (eg, Saudi Arabia).

Searches were limited to experimental reports found in journal articles published between January 1995 and July 2015. Limits for *humans* were flexibly applied in the electronic searches in PubMed and PsycINFO, though unindexed articles were captured by removing the *humans* filter from 2013 onwards. Details of the search strategy can be found in Multimedia Appendix 1.

In addition, we hand searched citations of eligible articles, and forward citation of protocol articles found in the search, in order to identify additional relevant published studies. Finally, at the third conference of the *European Society for Research on Internet Interventions* in Poland in September 2015, where we presented preliminary review results, we asked the audience of experts in e-mental health interventions to inform us if they were aware of any articles that we may have missed. All citations were managed using EndNote X7 (Thomson Reuters) and references and abstracts were exported into Microsoft Excel for easy title and abstract screening.

### Study Selection

The inclusion criteria applied to articles to be included in this study are shown in Textbox 1.

**Inclusion criteria for articles.**

Population:

- More than 75% of participants above a clinical cutoff for symptoms of unipolar depression or anxiety including trauma-related disorders, irrespective of the clinical measure used
- People culturally and linguistically different to those for which the intervention was originally designed

Intervention:

- A minimally-guided or unguided self-help program; one hour or less of face-to-face health worker or trained layperson time or up to 90 minutes total telephone or email support [8], regardless of delivery mode
- Structured and active therapeutic modality (ie, the intervention has clear theoretical underpinnings or an evidence base)
- Must include methodology used (ie, an observational or controlled study, process report, or a protocol)

Comparator:

- Any control condition, including placebo, treatment/care as usual, waitlist control, or active treatment comparison

Outcome:

- Postintervention measures of symptoms of mental illness

Study design:

- Randomized or nonrandomized experimental studies

**Exclusion criteria for articles.**

- Intervention(s) as an adjunct to traditional face-to-face therapy
- Delivered in an inpatient setting
- Intervention designed for the culturally and linguistically diverse (CALD) population, therefore not adapted
- Training materials for health workers
- Prevention programs for mental disorders

The exclusion criteria applied to articles to be excluded from this study are shown in Textbox 2.

Two researchers (EH and MHS) independently screened titles and abstracts according to the inclusion and exclusion criteria, which were then applied to the full texts of the eligible publications. All disagreements were discussed and resolved. Where it did not become clear from the full text, we wrote to investigators to verify the eligibility of the primary study, focusing in particular on whether the intervention was minimally guided and adapted for, not developed for, culturally and linguistically diverse populations.

### Data Extraction and Additional Data Collection

Two researchers (EH and MHS) designed and piloted a data extraction tool by considering all study characteristics related to the research question considering the PICO elements.

Consistent with the Cochrane Collaboration approach and the methods used in a recent review of cultural adaptations of traditional psychological interventions [24], we developed a structured checklist to critically appraise the methodological quality of the included studies considering the following four criteria: method of randomization, allocation concealment, blinding of outcome assessment, and attrition bias [24]. Two researchers (EH and MHS) independently applied the checklist to each of the included primary studies and a third researcher (EA) was called upon in cases of discordance of opinion.

In order to find out more about cultural adaptation methods used by researchers, we developed a short online questionnaire based on the framework by Bernal and Sáez-Santiago [19] (see Multimedia Appendix 2) and asked the authors of the included studies to complete it. Based on the information from the full-text articles and the questionnaires received, we assigned each study a score according to the number of Bernal and Sáez-Santiago framework adaptation elements that were applied.

### Data Analysis

We retained only the outcome measure designed as the primary endpoint in each study. We entered the number of participants, postintervention means and standard deviations, and the number of adaptation points into Stata 13 (StataCorp LP) [27]. Then we stratified the analyses by the adaptation score and conducted a meta-analysis using a random-effect method to calculate the pooled effect size based on the combination of the standardized mean differences of primary studies, and formally assessed and quantified heterogeneity using Higgins’ I^2^ [28]. We then examined whether the extent of cultural adaptation explained the heterogeneity across studies using an unadjusted random-effects meta-regression model.

In a sensitivity analysis, we tested the robustness of using primary outcomes data only for our main analysis by rerunning the meta-analysis separating depression and anxiety data, irrespective of whether measures were used as primary or secondary endpoints. We then compared the I^2^ values and 95% CIs between models to gain insight into the potential contribution of the primary versus secondary outcome to the heterogeneity observed in the stratified analyses.

## Results

### Systematic Search and Study Selection

We identified 4911 records, and excluded 1125 duplicates and 3585 citations on the basis of their titles and abstracts, which left 101 publications that were taken forward for full-text review. Of these, 11 were conference abstracts only, leaving 90 full texts to screen. Two articles were in Korean, so a Korean-speaking acquaintance of the research team was given guidance as to how to screen the articles, neither of which met inclusion criteria [29,30].

**Figure 1:**
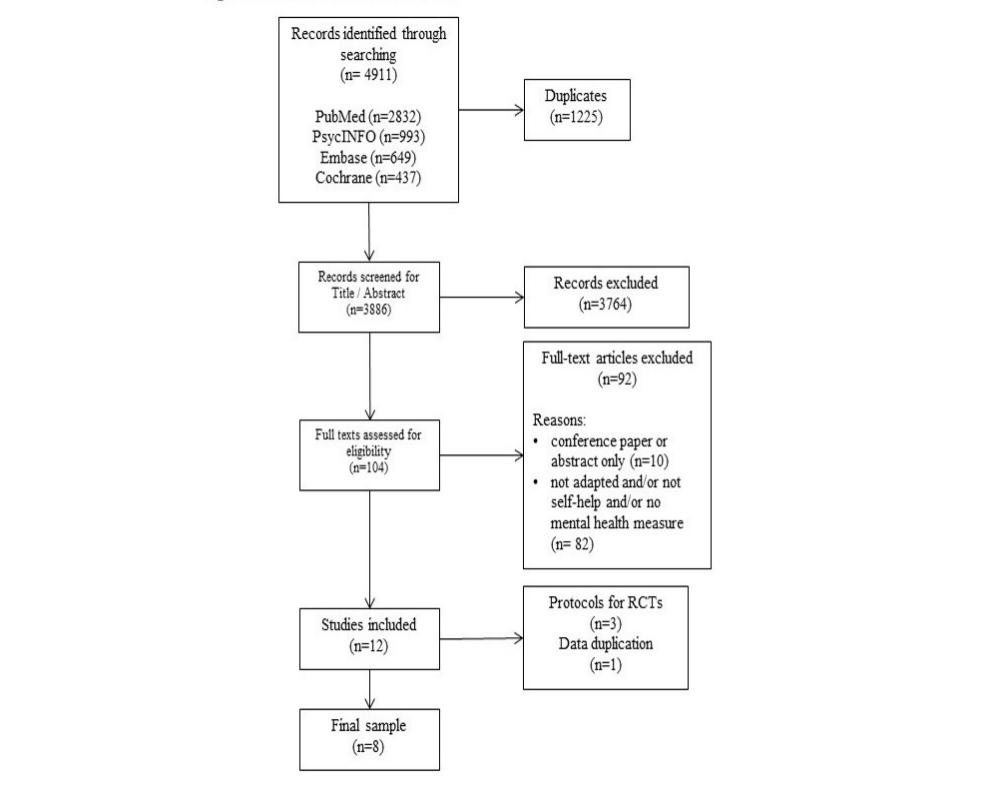
Preferred Reporting Items for Systematic Reviews and Meta-Analyses (PRISMA) diagram with systematic search and selection process. RCT: randomized controlled trial.

Reasons for exclusion are reported in Figure 1. Briefly, several articles did not meet various inclusion criteria, examples of which follow: they were not interventions designed in the West and adapted for a CALD population, but instead were designed specifically for the population [31-33]; the participants were not considered a CALD population [34-36]; the interventions were not self-help according to our standard definition above [37-39]; and/or no common mental disorder outcome measure was reported [40-42].

A total of 12 articles were retrieved (see PRISMA diagram in Figure 1); eight were randomized controlled trials (RCTs), two were protocols for included RCTs [43,44], and one was a protocol for an RCT whose results have not yet been published [45]. One additional article [46] utilized one of the datasets already included [47] (ie, urban sample). One of the included RCTs [47] had two different study sites—urban and rural—with different methodologies; therefore, we treated these as two different datasets. Four studies investigated the effect of bibliotherapy [47-50], and the remaining four were of e-mental health interventions [51-54].

One additional unpublished dataset was identified from key informants at the European Society for Research on Internet Interventions (ESRII) conference, but this was excluded. Upon contacting the author, there was insufficient information to determine whether the intervention was designed for the CALD population and, therefore, whether the study met our inclusion criteria. Characteristics of the studies are presented in Table 1.

There were eight RCTs, including nine datasets for analysis. The cultural backgrounds of the participants included in the studies were Chinese, Romanian, Pakistani, Japanese, and Turkish. Most of the interventions had a cognitive behavioral approach [48,50-52]; however, one study used a problem solving approach [44], one study with two datasets used a social cognitive theory approach [47], and one study used acceptance and commitment therapy [53]. The duration of interventions ranged from 4 to 12 weeks and only two out of eight interventions were completely self-help (ie, no guidance from a health worker) [53]. The number of Bernal and Sáez-Santiago framework adaptation points carried out ranged from 0 to 7, out of a possible 8. Two interventions, across three datasets, focused primarily on anxiety symptoms, and six on depressive symptomatology, with a range of outcome measures to quantify effects.

**Table 1:** Characteristics of RCT^a^ studies retrieved.

Study	Randomized, n (analyzed, n)	CALD^b^ group	Delivery	Therapy approach	Length (weeks)	Guidance	Adapt.^c^ points	Primary outcome measure
Choi et al 2012 [48]	63 (51)	Chinese	e-MH^d^	CBT^e^	8	MG^f^	7	(C)BDI II^g^ depression
Liu et al 2009 [51]	52 (40)	Chinese	Biblio.^h^	CBT	4	MG	0	(C)BDI II depression
Moldovan et al 2013 [52]	96 (84)	Romanian	Biblio.	CBT	4.5	MG	0	BDI II^i^ depression
Muto et al 2011 [53]	70 (42)	Japanese	Biblio.	ACT^j^	8	SH^k^	5	GHQ^l^ depression
Naeem et al 2014 [54]	192 (183)	Pakistani	Biblio.	CBT	12	MG	5	HADS-D^m^ depression
Tulbure et al 2015 [50]	76 (68)	Romanian	e-MH	CBT	9	MG	5	LSASSR^n^ anxiety
Ünlü Ince et al 2013 [49]	96 (56)	Turkish	e-MH	PS^o^	5	MG	5	CES-D^p^ depression
Wang et al 2013 [47] (urban)	103 (61)	Chinese	e-MH	SCT^q^	4.5	SH	3	PDS^r^ anxiety
Wang et al 2013 [47] (rural)	94 (90)	Chinese	e-MH	SCT	4.5	SH	3	PDS anxiety

^a^RCT: randomized controlled trial.

^b^CALD: culturally and linguistically diverse.

^c^adapt: adaptation.

^d^e-MH: e-mental health.

^e^CBT: cognitive behavioral therapy.

^f^MG: minimally guided.

^g^(C)BDI II: (Chinese) Beck Depression Inventory II.

^h^biblio.: bibliotherapy.

^i^BDI II: Beck Depression Inventory II.

^j^ACT: acceptance and commitment therapy.

^k^SH: self-help.

^l^GHQ: General Health Questionnaire.

^m^HADS-D: Hospital Anxiety and Depression Scale.

^n^LSASSR: Liebowitz Social Anxiety Scale Self Report.

^o^PS: problem solving.

^p^CES-D: Center for Epidemiological Studies Depression Scale.

^q^SCT: social cognitive theory.

^r^PDS: Patient Distress Scale.

### Data Extraction and Additional Data Collection

We determined the overall risk of bias of the included studies to be moderate. The main issue that introduced potential bias into studies was the outcome measures being subjective self-report measures, coupled with the fact that participants could not be blinded. Details on risk of bias assessment are reported in Multimedia Appendix 3.

**Figure 2:**
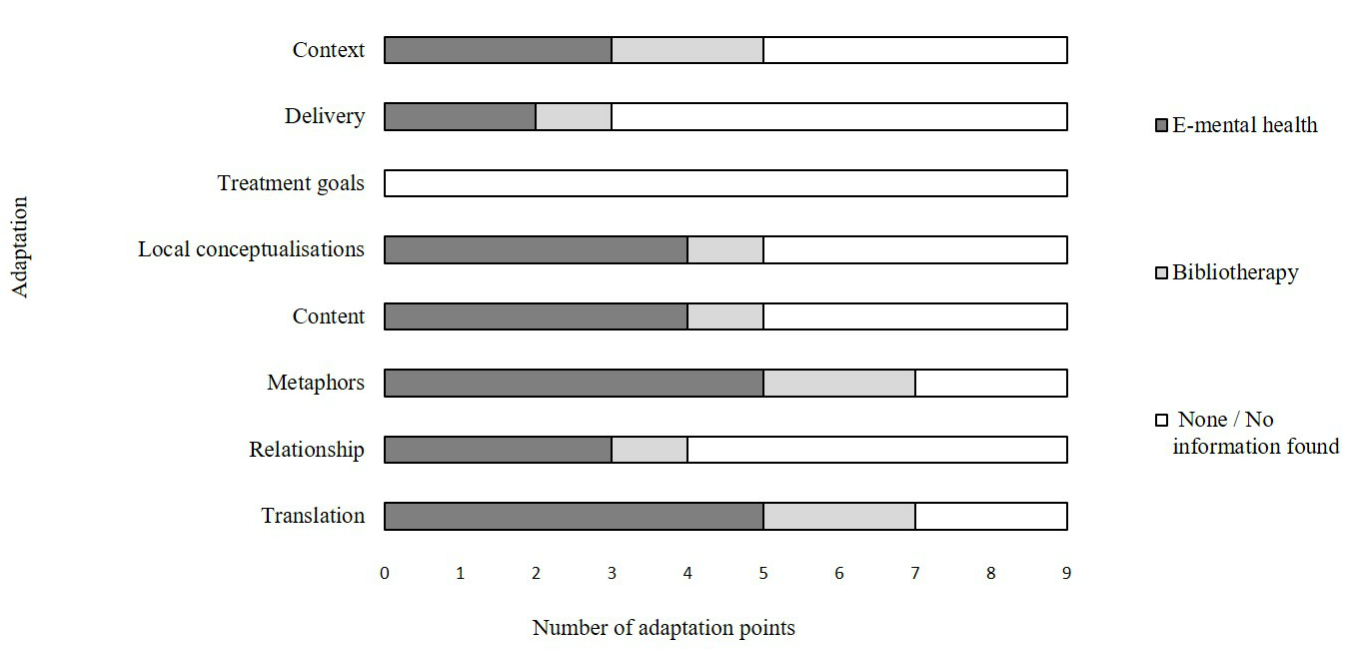
Adaptation score assigned to the selected studies.

The cultural adaptation methods used were only minimally reported in the primary publications. Six researchers responded to the questionnaire that we sent. Using questionnaire responses, where provided, and the full texts of the RCTs plus any protocols or related previously published studies, we assigned each RCT an adaptation score according to the number of Bernal and Sáez-Santiago framework adaptation elements that we deemed were applied (see Figure 2). In some cases, researchers had considered an element of adaptation but chosen not to carry out that adaptation having not identified a need for it. These were coded as affirmative responses; examples of affirmative questionnaire responses can be found in Multimedia Appendix 4.

All but two researchers indicated that they had translated their interventions into the language of the target group. Seven researchers—five in e-mental health interventions—reported the use of adapted metaphors, using symbols, concepts, idioms, and sayings from the target culture. Local values and traditions in order to carry the content of the intervention were considered by five researchers—four in e-mental health interventions. Theoretical concepts and constructs were considered by four e-mental health researchers and one bibliotherapy researcher. No researcher reported having considered treatment goals in the adaptation of their intervention. The delivery method of the intervention (eg, making particular allowances or breaking strategies into smaller tasks) was mentioned in the questionnaire response of three researchers—two e-mental health interventions. The socioeconomic and political context of the intervention was considered by five researchers—two e-mental health interventions—yet most researchers who responded to the qualitative element of this question mentioned that e-mental health or bibliotherapy in itself was used in response to the socioeconomic and cultural environment (eg, stigma and access to services).

### Data Analysis

**Figure 3:**
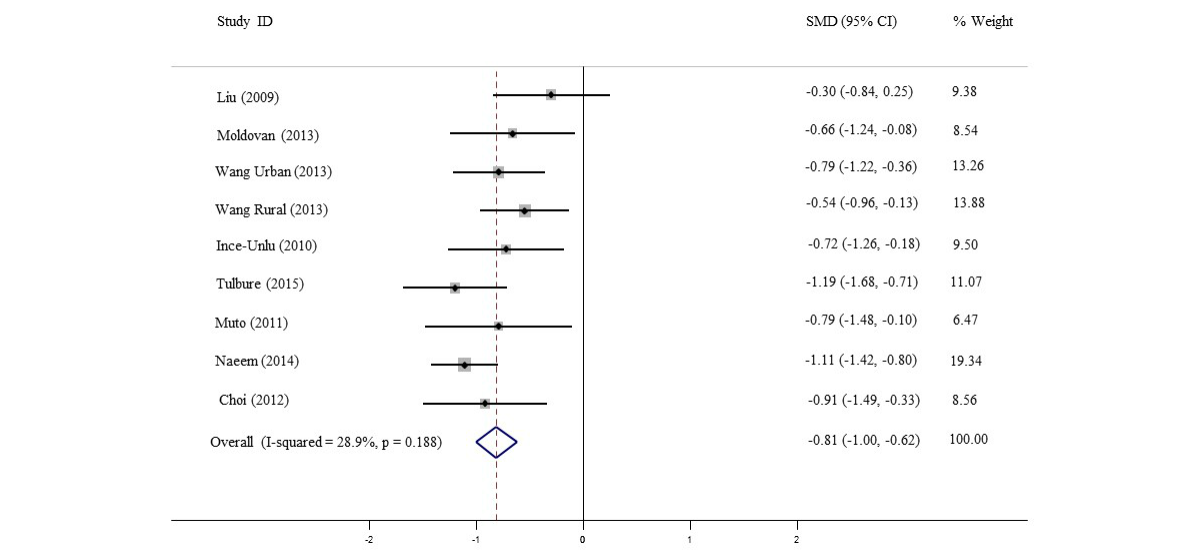
Random-effects meta-analysis of primary outcome measures in order of the study adaptation score. SMD: standardized mean difference.

The random-effects meta-analysis (see Figure 3) on the primary outcome measures included nine datasets with a total of 684 participants. We stratified the data by descending assigned cultural adaptation points.

Overall, the minimally guided and self-help interventions significantly improved depression and anxiety symptomatology; the pooled standardized mean difference (SMD) from primary outcome measures was -0.81 (95% CI -0.10 to -0.62), with low-to-moderate between-studies heterogeneity (I^2^=28.9%).

**Figure 4:**
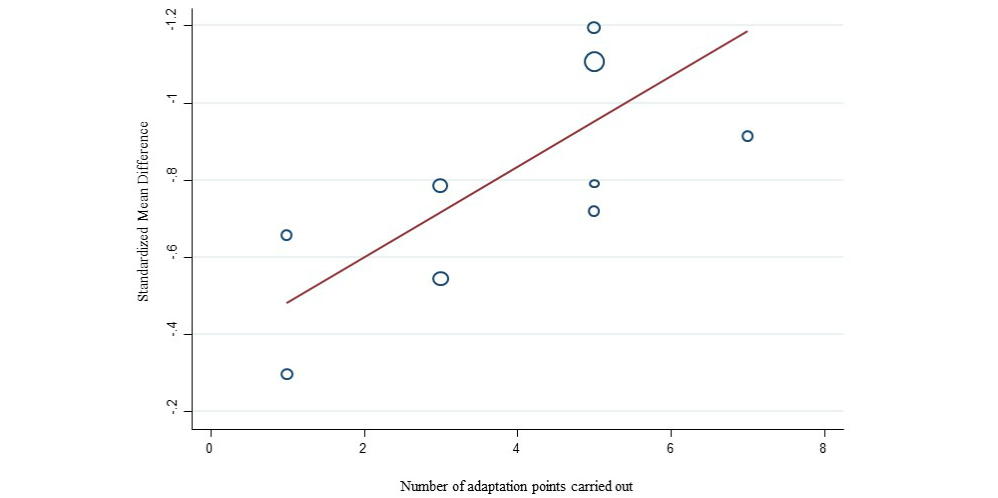
Meta-regression of the standardized mean difference and adaptation score.

The meta-regression (see Figure 4) showed that the adaptation scores significantly explained the pooled SMD. Specifically, a 1-point increase in the adaptation score was significantly associated with an increase in effect size of 0.117 (*P*=.04), or a 14% rise in pooled efficacy.

To test the robustness of our main model, which focused on the primary outcome measures, we carried out two separate meta-analyses for each outcome (ie, depressive or anxiety symptoms), irrespective of what the intervention was primarily designed for. Our results were largely unchanged, but the heterogeneity across the studies was greater (SMD=-0.65 [95% CI -0.92 to -0.38], I^2^=62.2% for anxiety and SMD=-0.58 [95% CI -0.93 to -0.24], I^2^=75.4% for depression), thus confirming our use of primary measures only in the analysis.

## Discussion

### Principal Findings

Culturally adapted self-help or minimally guided bibliotherapy and e-mental health interventions moderately, but significantly, reduced depressive and anxious symptomatology in populations culturally and linguistically distinct from those for which the interventions had been originally designed. More extensive cultural adaptation of the interventions under study was significantly associated with larger effect sizes; however, details of the cultural adaptation methodologies applied were largely underreported.

Researchers should be encouraged to report in detail their adaptation methods to enable readers to appraise both internal and external validity of the study findings, and to inform the implementation of minimally guided interventions and their scalability beyond the research context.

Our results have both public health and clinical relevance, particularly to program managers and health workers who aim to use minimally guided interventions among CALD clients. Decision makers should consider the implications of cultural adaptation on the expected efficacy of interventions before use and, if feasible, plan the required resources and time investments accordingly. A 14% rise in efficacy per adaptation point increase should be weighed against the costs of adapting intervention content, considering the anticipated coverage of the intervention. Adaptation could also positively affect attrition rates, which can be high in self-help interventions.

Our results suggest that the effect of minimally guided interventions used in CALD populations was significantly influenced by cultural adaptation. We were unable to review the specifics of qualitative methodologies involved, but depending on resources, adaptation could resemble a single focus group of community members, or a costly and extensive multi-stakeholder consultation program. Precise information on the methods used is important to allow, on the one hand, a comprehensive adaptation to other settings and contexts and, on the other hand, the preservation of the original intervention’s core therapeutic techniques and components to maximize the fidelity to the original intervention.

The Bernal and Sáez-Santiago framework was extremely useful but difficult to operationalize. Further research is needed to develop, pilot, and test an adaptation protocol for minimally guided interventions that would favor the utility and efficiency of the original framework’s use in cross-cultural psychology and psychiatry. Such an adaptation protocol should strive to include the target community in qualitative research and discussions on adaptation of materials.

### Limitations

Our study has limitations. First, we focused on selected countries that we arbitrarily considered to be culturally distinct. However, this choice was extensively discussed to include geographically diverse LMICs and high-income countries culturally diverse from North America, Australia, and Western Europe, where the majority of minimally guided interventions are developed and tested. We did not consider potential cultural differences between Western countries (eg, an intervention designed in the United States and used in Norway), and we did not focus on indigenous populations as culturally diverse, and underserved, groups.

Second, and briefly mentioned above, although the meta-analysis showed low heterogeneity across the studies, the meta-regression used to formally explore this further was based on only nine datasets for a total of 684 participants. The Cochrane handbook suggests a minimum of 10 studies to run a meta-regression [55], so these findings should be considered with caution. Further, because half of the studies (n=5) included completers only in their analyses, the reported effect sizes might have been inflated, and an overestimation of the true effect in our meta-analysis cannot be excluded [56]. In addition, the marked differences in intervention duration and in follow-up times between studies may further limit comparability. In the meta-regression, we focused only on the number of Bernal adaptation elements. This was coherent with our main scope and was statistically appropriate (see above). However, other study design characteristics could also explain the between-studies heterogeneity and, at least to some extent, they might even confound or modify the observed effect of cultural adaptation.

Third, using the Bernal and Sáez-Santiago framework carries its own limitations. It was developed over 20 years ago in North America in relation to transcultural issues of working with Latino communities. In addition, it was informed by a theory-driven and anecdotal approach, as opposed to being informed by community-based explorative and qualitative data. We used the Bernal and Sáez-Santiago framework because it had been used to categorize adaptations made in previous systematic reviews [22-24]. The number of elements of the Bernal and Sáez-Santiago framework carried out in each study was based on a subjective assessment of the information from full texts and the qualitative information provided in the questionnaires. Though authors agreed that they had carried out most adaptation elements, the review researchers independently tended to assign lower completion rates. This coding remained subjective on both the authors’ and the primary study researchers’ part, mainly because Bernal concepts are rather abstract and can sometimes overlap. Also, if researchers stated that they had considered an element but chosen not to carry it out, we coded this as an affirmative answer. A further limitation of the adaptation coding stemmed from the fact that the Bernal and Sáez-Santiago framework was developed for face-to-face treatments. We chose to use the framework in its original state. Some of the categories (ie, treatment goals or therapeutic relationship) are likely less or modestly relevant for minimally guided interventions.

### Comparison With Prior Work

Our results are consistent with evidence from previous systematic reviews on the cultural adaptation of face-to-face interventions for common mental disorders [21-24], particularly that completing more elements of adaptation is associated with a higher effect size [22]. Indeed, the effect size of the most comprehensively adapted, Chinese version of the *Sadness* intervention [48] was similar, if not slightly higher, compared to its original Australian version [57]—within-groups Cohen’s d for reduction in Beck Depression Inventory score was 1.41 in the Chinese adapted intervention and 1.27 in the original Australian version.

Further comparisons with previous findings are less straightforward. Statistical power was limited by the total number of studies included (see below), which were not sufficient to conduct advanced, multivariable, meta-regression models to test which, if any, of the Bernal *adaptation elements* might have contributed the most to the observed between-studies heterogeneity. Similarly, we were not able to consider other plausibly relevant covariates in our models, such as therapeutic modality, level of health worker support, length of delivery, and level of engagement or medium. Evidence on the impact of cultural adaptation is scant, but previous studies found that therapeutic goals, metaphors and symbols [22], and conceptualizations (ie, explanatory models) [21] may significantly account for variance in effect sizes of the interventions under study. These previous findings seem plausible because, although little is known about specific components that determine the effectiveness of a self-help program [9], providing users with an explanatory model of their distress using meaningful terms and symbols may constitute a critical prerequisite of minimally guided interventions.

### Conclusions

In conclusion, our results support the careful application of cultural adaptation of minimally guided and self-help interventions, whether provided via bibliotherapy or the Internet, before their use in diverse settings and populations. This largely applies to the *globalization* of mental health services and psychological interventions, for which cultural adaptation is key. Further, there is also a moral case to test and demonstrate the appropriateness, acceptability, and harmlessness of interventions up front. Cultural adaptation is explicitly intended to render interventions meaningful and helpful to groups that are culturally diverse from those for which the intervention was designed. Therefore, these findings may be particularly relevant to program managers and treatment providers in non-Western settings.

## Appendix

Multimedia Appendix 1 Search strategy.

Multimedia Appendix 2 Questionnaire for researchers.

Multimedia Appendix 3 Risk of bias analysis.

Multimedia Appendix 4 Example responses from researchers.

## Abbreviations

ACT: acceptance and commitment therapy
adapt.: adaptation
BDI II: Beck Depression Inventory II
biblio.: bibliotherapy
CALD: culturally and linguistically diverse
(C)BDI II: (Chinese) Beck Depression Inventory II
CBT: cognitive behavioral therapy
CES-D: Center for Epidemiological Studies Depression Scale
e-MH: e-mental health
ESRII: European Society for Research on Internet Interventions
GHQ: General Health Questionnaire
HADS-D: Hospital Anxiety and Depression Scale
HIC: high-income country
iCBT: Internet cognitive behavioral therapy
LMICs: low- and middle-income countries
LSASSR: Liebowitz Social Anxiety Scale Self Report
MG: minimally guided
PDS: Patient Distress Scale
PICO: population, intervention, comparator, and outcomes
PRISMA: Preferred Reporting Items for Systematic Reviews and Meta-Analyses
PS: problem solving
RCT: randomized controlled trial
SCT: social cognitive theory
SH: self-help
SMD: standardized mean difference
WHO: World Health Organization
