# Supplementary material for: Cultural Adaptation of Minimally Guided Interventions for Common Mental Disorders: A Systematic Review and Meta-Analysis
Source: JMIR Ment Health. 2016 Sep 26;3(3):e44. doi: 10.2196/mental.5776 (PMC5057065; doi:10.2196/mental.5776)
Supplement: Multimedia Appendix 1 [file mental_v3i3e44_app1.pdf]

Appendix 1. Search strategy

We started by writing a generic search strategy, that was slightly revised for the different databases depending on their indexing terms and medical subheadings system. Guidance was sought from professional librarians where a search concept was problematic. The example systematic search below was that used for pubmed.

Search strategy mode of delivery:

“mobile applications” [MeSH] OR “cell phone” [MeSH] OR telemedicine [MeSH] OR Internet [TIAB] OR multimedia [TIAB] OR multi-media [TIAB] OR online [TIAB] OR computer [TIAB] OR computerized [TIAB] OR computerised [TIAB] OR phone [TIAB] OR smartphone [TIAB] OR Smartphones [TIAB] OR smart-phone [TIAB] OR “web based” [TIAB] OR webbased [TIAB] OR web-based [TIAB] OR “electronic health” [TIAB] OR e-health [TIAB] OR eHealth [TIAB] OR telecare [TIAB] OR telemedicine OR telehealth OR tele-health [TIAB] OR “mobile care” [TIAB] OR m-health [TIAB] OR mHealth [TIAB] OR “mobile-health” [TIAB] OR “mobile health” [TIAB] OR e-mail [TIAB] OR email [TIAB] OR virtual [TIAB] OR cd [TIAB] OR cd-rom [TIAB] OR dvd [TIAB] OR game [TIAB] OR software [TIAB] OR audio [TIAB] OR audiovisual [TIAB] OR video [TIAB] OR media-based [TIAB] OR hypermedia [TIAB] OR ((App) NOT amyloid) NOT atrial) OR apps [TIAB] OR bibliotherapy [TIAB] OR “self-help book” [TIAB]

Search concept intervention:

“self help” [TIAB] OR self-help [TIAB] OR “self change” [TIAB] OR self-change [TIAB] OR self-care [TIAB] OR selfcare [TIAB] OR self-management [TIAB] OR “self directed” [TIAB] OR self-direct* [TIAB] OR self-admin* [TIAB] OR self-instructed [TIAB] OR self-instructional [TIAB] OR self-instruction [TIAB] OR e-therap* [TIAB] OR cCBT [TIAB] OR iCBT OR “minimally guided” [TIAB] OR “minimal guidance” [TIAB] OR therapy [TIAB] OR therapies [TIAB] OR treatment [TIAB] OR intervention [TIAB] OR advice [TIAB] OR program* [TIAB] OR counselling [TIAB] OR counseling [TIAB] OR psychoeducation [TIAB] OR skills [TIAB]

Search concept Common Mental Disorders

Filter 20 years, human:

“Behavioural symptoms” [MeSH] OR “Affective symptoms” [MeSH] OR “Adjustment Disorders” [MeSH] OR “Anxiety Disorders” [MeSH] OR “psychological stress” [MeSH] OR “Obsessive-Compulsive Disorder” [MeSH] OR “Panic Disorder” [MeSH] OR “Phobic Disorders” [MeSH] OR “Stress Disorders” [MeSH] OR “Mood Disorders” [MeSH] OR “Affective Disorders” [MeSH] OR “Depressive Disorder”

OR mental [TIAB] OR Adjustment [TIAB] OR Affective [TIAB] OR Anxiety [TIAB] OR post-traumatic [TIAB] OR Panic [TIAB] OR Phobic [TIAB] OR phobia [TIAB] OR Stress [TIAB] OR Mood [TIAB] OR “post-traumatic” [TIAB] OR Affective [TIAB] OR depression [TIAB] OR depressive [TIAB] OR distress [TIAB]

Search concept LAMIC (+ a few other culturally diverse HIC):

Afghanistan OR Albania OR Algeria OR Angola OR Antigua OR Barbuda OR Argentina OR Armenia OR Armenian OR Aruba OR Azerbaijan OR Bahrain OR Bangladesh OR Barbados OR Benin OR Byelarus OR Byelorussian OR Belarus OR Belorussian OR Belorussia OR Belize OR Bhutan OR Bolivia OR Bosnia OR Herzegovina OR Hercegovina OR Botswana OR Brasil OR Brazil OR Bulgaria OR Burkina OR Faso OR Fasso OR Volta OR Burundi OR Urundi OR Cambodia OR Khmer OR Kampuchea OR Cameroon OR Cameroons OR Cameron OR Camerons OR Cape Verde OR African OR Chad OR Chile OR China OR Colombia OR Comoros OR Comoro OR Comores OR Mayotte OR Congo OR Zaire OR Rica OR d’Ivoire OR Ivory OR Cuba OR Djibouti OR Somaliland OR Dominica OR Dominican OR Timor OR Timur OR Leste OR Ecuador OR Egypt OR Arab OR Salvador OR Eritrea OR Ethiopia OR Fiji OR Gabon OR Gabonese OR Gambia OR Gaza OR Georgia OR Georgian OR Ghana OR Grenada OR Guatemala OR Guinea OR Guam OR Guiana OR Guyana OR Haiti OR Honduras OR Hungary OR India OR Maldives OR Indonesia OR Iran OR Iraq OR Jamaica OR Jordan OR Kazakhstan OR Kazakh OR Kenya OR Kiribati OR Korea OR Kosovo OR Kyrgyzstan OR Kirghizia OR Kyrgyz OR Kirghiz OR Kirgizstan OR Lao OR Laos OR Lebanon OR Lesotho OR Basutoland OR Liberia OR Libya OR Macedonia OR Madagascar OR Malagasy OR

Malaysia OR Malaya OR Malay OR Sabah OR Sarawak OR Malawi OR Nyasaland OR Mali OR Marshall OR Mauritania OR Mauritius OR Agalega OR Mexico OR Micronesia OR Moldova OR Moldovian OR Mongolia OR Montenegro OR Morocco OR Ifni OR Mozambique OR Myanmar OR Myanma OR Burma OR Namibia OR Nepal OR Antilles OR Caledonia OR Nicaragua OR Niger OR Nigeria OR Mariana OR Oman OR Muscat OR Pakistan OR Palau OR Palestine OR Palestinian OR Panama OR Paraguay OR Peru OR Philippines OR Philipines OR Phillipines OR Phillippines OR Filipino OR Puerto Rico OR Romania OR Rumania OR Roumania OR Russia OR Russian OR Rwanda OR Ruanda OR Kitts OR Nevis OR Lucia OR Vincent OR Grenadines OR Samoa OR Samoan OR Navigator OR Sao Tome OR Saudi Arabia OR Senegal OR Serbia OR Montenegro OR Seychelles OR Sierra OR Leone OR Sri OR Lanka OR Ceylon OR Solomon OR Somalia OR Africa OR Sudan OR Suriname OR Surinam OR Swaziland OR Syria OR Tajikistan OR Tadzhikistan OR Tadjikistan OR Tadzhik OR Tanzania OR Thailand OR Togo OR Togolese OR Tonga OR Trinidad OR Tobago OR Tunisia OR Turkey OR Turkmenistan OR Turkmen OR Uganda OR Ukraine OR Uruguay OR USSR OR Soviet OR Uzbekistan OR Uzbek OR Vanuatu OR Hebrides OR Venezuela OR Vietnam OR Viet Nam OR West-Bank OR Yemen OR Yugoslavia OR Zambia OR Zimbabwe OR Rhodesia OR Polynesia OR Hong Kong OR Isreal OR Macao OR Macau OR Qatar OR Singapore OR Emirates OR Afghan OR Albanian OR Algerian OR Angolan OR Antiguan OR Barbadian OR bajun OR Argentinean OR Armenian OR Aruban OR Azerbaijani OR Aziri OR Bahraini OR Bangladeshi OR Beninese OR Belarussian OR Belizean OR Bhutanese OR Bolivian OR Bosnian OR Herzegovinian OR Batswana OR Brazilian OR Bulgarian OR Burkinabe OR Burundian OR Cambodian OR Khmer Cameroonian OR ‘Cape Verdian’ OR African OR Chadian OR Chilean OR Chinese OR Colombian OR Comoran OR Mahorais OR Congolese OR Ivoirian OR Cuban OR Djibouti OR Somali OR somalian OR Dominican OR Atoni OR Ecuadorian OR Egyptian OR Arab OR Salvadorian OR Eritrean OR Ethiopian OR Fijians OR Gabonese OR Gambian OR Georgian OR Ghanaian OR Grenadian OR Guatemalan OR Guinean OR Guamanian OR chamorro OR Guyanese OR Haitian OR Honduran OR Hungarian OR Indian OR Maldivians OR Indonesian OR Iranian OR Iraqi OR Jamaican OR Jordanian OR Kazakh OR Kenyan OR Kiribati OR Korean OR Kosovar OR Kyrgyzstani OR Kyrgyz OR Laotian OR Latino OR Lebanese OR Basotho OR Liberian OR Libyan

OR Macedonian OR Malagasy OR Malaysian OR Malay OR Malawian OR Malian OR Marshallese OR Mauritanian OR Mauritian OR Mexican OR Micronesian OR Moldovan OR Mongolian OR Montenegrin OR Moroccain OR Mozambican OR Myanmarese OR Burmese OR Namibian OR Nepalese OR Antilles OR Caledonians OR Nicaraguan OR Nigerian OR Omani OR Pakistani OR Palauan OR Palestinian OR Panamanians OR Paraguayan OR Peruvian OR Filipino Philippino OR 'Puerto Rican' OR Romanian OR Russian OR Rwandan Lucian OR Samoan OR Navigator OR 'Sao Tomean' OR Saudi OR Arabian OR Senegalese OR Serbian OR Seychellois OR 'Sierra Leonean' OR 'Sri Lankan' OR Solomon OR African OR Sudanese OR Surinen OR Swazi OR Syrian OR Tajiks OR Tanzanian OR Thai OR Togolese OR Tonga OR Trinidadian OR Tobagonian OR Tunisian OR Turkish OR Turkmenistani OR Turkmen OR Ugandan OR Ukrainian OR Uruguayan OR Uzbek OR Vanuatu OR Venezuelan OR Vietnamese OR Yemeni OR Yugoslavian OR Zambian OR Zimbabwean OR Rhodesian OR Polynesian OR 'Hong Kongese' OR Israeli OR Macanese OR Qatari OR Singaporean OR Emirati
