# Supplementary material for: Cultural Adaptation of Minimally Guided Interventions for Common Mental Disorders: A Systematic Review and Meta-Analysis
Source: JMIR Ment Health. 2016 Sep 26;3(3):e44. doi: 10.2196/mental.5776 (PMC5057065; doi:10.2196/mental.5776)
Supplement: Multimedia Appendix 2 [file mental_v3i3e44_app2.pdf]

Appendix 2: Questionnaire sent to researchers on methodology

Dear Colleague,

Many thanks for completing this survey, which will take you approximately 10-15 minutes, depending on the amount of information you wish to provide. Please let us know if you prefer a short telephone interview, in this case, we are happy to call you.

We would like to thank you very much in advance for your help on this important information gathering exercise.

Best wishes,

Melissa Harper and Eva Heim

(If you would like to know more about the design of this survey, it was informed by the following references:

Chowdhary, N., Jotheeswaran, A. T., Nadkarni, A., Hollon, S. D., King, M., Jordans, M. J., . . . Patel, V. (2014). The methods and outcomes of cultural adaptations of psychological treatments for depressive disorders: a systematic review. Psychol Med, 44(6), 1131-1146. doi: 10.1017/s0033291713001785

Bernal, G., & Sáez-Santiago, E. (2006). Culturally centered psychosocial interventions. Journal of Community Psychology, 34(2), 121-132. doi: 10.1002/jcop.20096)

Questions

Question 1. Please tell us more about how you adapted the intervention. Did you adapt the intervention in any of the following ways:

	**Yes**	**No**	**N/A**
1. Did you translate the intervention to local language?	☐	☐	☐
2. If applicable, did you consider culture in the patient-therapist relationship (e.g., the impact of cultural similarities or differences on therapeutic interactions or disclosures)?	☐	☐	☐
3. Did you consider metaphors in the cultural adaptation of your intervention (e.g., culturally relevant materials, stories, examples or symbols)?	☐	☐	☐
4. Did you adapt the content of the intervention to the local	☐	☐	☐

environment (e.g., focus on locally relevant stressors, values or customs, incorporation of local practices or additional material to account for local culture)?			
5. Did you adapt the intervention to account for local conceptualisations/idioms of distress of the presenting problem (e.g., somatisation, social roles and norms, spiritual beliefs around the problem)?	☐	☐	☐
6. Did you attempt to consider treatment goals in relation to cultural knowledge (e.g., treatment aims in relation to values, customs, and traditions)?	☐	☐	☐
7. Did you adapt the method of delivery or presentation of materials to the local culture (e.g. adapting ethnicity of illustrated people or using spoken word or pictures instead of text-heavy presentations)?	☐	☐	☐
8. Did you adapt to the broader social, economic, and political context of the user (e.g., consideration of accessibility, feasibility and acceptability of the intervention)?	☐	☐	☐

Question 2. We would very much appreciate if you could kindly describe the cultural adaptation methods you used for each of the previous questions you marked with "yes".

(Only displayed for methods that were marked before)

1. How did you translate the intervention to local language?
2. How did you consider culture in the patient-therapist relationship?
3. How did you consider metaphors in the cultural adaptation of your intervention (e.g., culturally relevant materials, stories, examples or symbols)?
4. How did you adapt the content of the intervention to the local environment (e.g., focus on locally relevant stressors, values or customs, incorporation of local practices or additional material to account for local culture)?
5. How did you adapt the intervention to account for local conceptualisations of the presenting problem (e.g., somatisation, social roles and norms, spiritual beliefs around the problem)?
6. How did you attempt to consider treatment goals in relation to cultural knowledge?
7. How did you adapt the method of delivery or presentation of materials to the local culture?
8. How did you adapt to the broader social, economic, and political context of the user (e.g., consideration of accessibility, feasibility and acceptability of the intervention)?

Question 3. For the development and evaluation of complex interventions, the Medical Research Council (in the UK) recommends a phased development process consisting of: modeling/theoretical development, formative work, piloting and evaluation.

(http://www.ncbi.nlm.nih.gov/pubmed/18824488).

We are interested at what stage of your study you possibly applied cultural adaptation methods. Thank you very much for kindly providing us with any relevant information on these four phases below.

Modelling/theoretical development (e.g., Identifying or developing theory, modelling processes and outcomes)	
Formative research (e.g., cultural research on the target population of the intervention)	
Pilot or feasibility testing (e.g., testing procedures, estimating recruitment and retention, Determining sample size)	
Evaluation (please describe the design of the evaluation research carried out or planned)	

Question 4. If you have any other comments or information you would like to provide, please add it here:
