# Supplementary material for: Cultural Adaptation of Minimally Guided Interventions for Common Mental Disorders: A Systematic Review and Meta-Analysis
Source: JMIR Ment Health. 2016 Sep 26;3(3):e44. doi: 10.2196/mental.5776 (PMC5057065; doi:10.2196/mental.5776)
Supplement: Multimedia Appendix 3 [file mental_v3i3e44_app3.pdf]

Appendix 3. Risk of bias of individual studies

	Random sequence	Allocation concealment	Blinding of outcome	Incomplete outcome
Choi 2012	[illegible]	[illegible]	[illegible]	[illegible]
Liu 2008	?*	?	[illegible]	[illegible]
Moldovan 2013	[illegible]	[illegible]	[illegible]	[illegible]
Muto 2011	[illegible]	[illegible]	[illegible]	[illegible]
Naeem 2014	[illegible]	?	[illegible]	[illegible]
Tulbure 2015	[illegible]	[illegible]	[illegible]	[illegible]
Unlu Ince 2013	?*	[illegible]	[illegible]	[illegible]
Wang Urban 2014	[illegible]	?	[illegible]	[illegible]
Wang Rural 2013	[illegible]	?	[illegible]	[illegible]

*Randomisation was reported but with no indication of how it was carried out
