# Supplementary material for: Cultural Adaptation of Minimally Guided Interventions for Common Mental Disorders: A Systematic Review and Meta-Analysis
Source: JMIR Ment Health. 2016 Sep 26;3(3):e44. doi: 10.2196/mental.5776 (PMC5057065; doi:10.2196/mental.5776)
Supplement: Multimedia Appendix 4 [file mental_v3i3e44_app4.pdf]

Appendix 4: Examples of qualitative questionnaire responses from researchers

1) Relationship with the therapist:

"This was an online depression program so there was minimal direct contact between patient and therapist. Patients were contacted by the therapists weekly to check in on progress, answer any questions, and provide encouragement. During telephone contact with the Chinese participants, the clinicians provided gentle encouragements and reinforced their effort in practicing homework, in contrast to giving explicit praise and reinforcement when working with Western participants."

2) Metaphors:

"- culturally specific idioms known as □□ Chéngyŭ (four-worded sayings derived from ancient stories or poems that deliver a moral teaching, and forms part of everyday Chinese vocabulary) were used to enhance meaning, e.g. to describe different types of negative thinking styles."

3) Content:

"…explicitly discussing migration and culture by using culture-specific cases and problems that are recognizable for the target group concerned, and including recognizable examples of persons with similar problems (eg, a young woman who migrated 2 years ago and can't find her way in the Netherlands)."

4) Theoretical concepts and constructs:

"No modifications were felt to be necessary in the central concepts of ACT to fit a Japanese reader—the issue was the means of presenting those ideas."

"The term depression itself hasn't been used in the intervention. In fact, all other symptoms of depression were used to make it more recognizable and less stigmatizing for the target group."

5) Delivery method:

"…modified exercises…to fit the Japanese language community."

"Each participant is allowed to choose his/her language of preference."

6) Socio-economic and political context:

“[The book] can be used by the patients or carers with at least 5 years of education.”

“Due to lack of Internet service at home in Beichuan, all participants had to complete tests and receive online treatment in the counseling center’s computer room.”
